# Quality Of Life in Children With Home Mechanical Ventilation – A Scoping Review

**DOI:** 10.1177/23779608221094522

**Published:** 2022-04-26

**Authors:** Janet Mattson, Johan Lunnelie, Tim Löfholm, Elina Scheers Andersson, Ragnhild E. Aune, Gunilla Björling

**Affiliations:** 1 Department of Health Sciences, Swedish Red Cross University, Sweden; 2 Department of Learning, Informatics, and Medical Education, 411412 Karolinska Institutet, Sweden; 3 Department of Material Science, 8018 Norwegian University of Science and Technology, Norway; 4 Department of Neurobiology, Care Sciences and Society, 411412 Karolinska Institutet, Sweden; 5 108094 Kilimanjaro Christian Medical University College, Faculty of Nursing, Tanzania

**Keywords:** adolescents and children, home mechanical ventilation, parent-proxy, quality of life, respiratory insufficiency

## Abstract

**Introduction:**

Home mechanical ventilation is an established method to support children suffering from chronic respiratory insufficiency, still more research is needed regarding mechanically ventilated children's and adolescents’ quality of life (QoL). Therefore, the aim of this scoping review was to explore research regarding QoL and lived experience of children and adolescents with home mechanical ventilation.

**Methods:**

A scoping review with systematic searches for research studies published between year 2000–2020 was performed in Cinahl, Medline, and PubMed. Studies that met the inclusion criteria were quality assessed and a thematic analysis was performed.

**Results:**

In total, ten articles were quality assessed and included in the results. Four themes emerged: Children's self-reported QoL, Parents’ perception and parent-proxy report, Differences between the child's and parent's perception, and challenges in daily life. Children with home mechanical ventilation reported a lower QoL than healthy children and children with other chronic diseases. Generally, parents rate their child's QoL lower than the children themselves.

**Conclusion:**

This is the first literature review focusing on HMV in the paediatric population. It is clear that HMV does not only affect the treated child or adolescent but also the whole family. It is important to regularly measure and evaluate QoL in children and adolescents with HMV to provide person-centered care. More research is needed to improve these children's and adolescents’ QoL.

## Introduction

### Growing Practice of Home Mechanical Ventilation in Children

Children dependent on mechanical ventilation are often treated during prolonged hospitalizations at the pediatric intensive care unit (PICU) (Ottonello et al., 2007). Technological advancements have increased the survival rate of these children (Ottonello et al., 2007; Redouane et al., 2016) and they can now live at home with home mechanical ventilation (HMV). Over the past decades, HMV is an established method for children suffering from chronic respiratory insufficiency (CRI) (Ottonello et al., 2007; Sterni, Collaco, Baker et al., 2016; Pekcan et al., 2010; Preutthipan, 2015; Redouane et al., 2016; Reiter et al., 2011). There are two kinds of HMV, Invasive (IV), ventilation by a tracheostomy tube, and Non-Invasive, (NIV), ventilation by a full-face or nasal mask. The choice of HMV is based on severity of the condition/underlying disease, indication, and physiological aspects (Laub, Berg & Midgren, 2006). Non-invasive ventilation is more frequently used and is the first choice of ventilatory support in HMV (Laub, Berg & Midgren, 2006). The choice of ventilation and type of ventilation must be individualized, where the underlying disease and condition should be considered. Regarding both IV and NIV, the fit of the tube or mask is very important. A tracheostomy tube should be properly fitted for an optimal comfort and function in order to minimize complications and give a sufficient airflow (Björling, 2009). The mask should minimize possible air leakage and be comfortable for the patient. A poorly fitted mask reduces the effectiveness of the ventilation (Reiter et al., 2011).

### HMV in Children

The first decision towards HMV is often taken in advanced clinics, such as PICUs or units specialized in respiratory diseases (Amirnovin, Aghamohammadi, Riley, Woo, & Del Castillo, 2018). There is no specific data on how many children receive HMV (Chatwin et al., 2015; Chau et al., 2017; Garner et al., 2013; Wallis et al., 2011), but there is an increase in high-income countries (Israelsson-Skogsberg, 2019). Preutthipan (2015) argues that it is challenging to initiate HMV in children since they have smaller airways. HMV can be challenging for caregivers and parents due to e.g. management of ventilators and medical devices, secretion mobilization and nightly support (Reiter et al., 2011). Children with HMV are dependent on their caregiver or family and of financial support from i.e., the government, healthcare insurance and must be considered before a child is discharged after initiation of HMV. A careful plan and education of caregivers and families are essential for safety reasons to minimize stress among family caregivers (Ottonello et al., 2007). HMV in children also require easy access to technical support and education to maintain patient safety (Chau et al., 2017).

### Patient- and Family Centered Care

The child's participation in daily activities is dependent on support from the health care system, the progression of the underlying disease or type of HMV (Israelsson, 2019). Preutthipan et al. (2015) highlights that difference in financial support depending on the healthcare system in a specific country has implications on the care of children with HMV. The increase of children with HMV calls for new care models including family centered care. The Institute of Patient- and Family-Centered Care declares patient- and family-centered care (PFCC) as an approach to plan, distribute, and evaluate health care. The PFCC has improved care, health outcome, care experience for patients and families, increased satisfaction among healthcare professionals (HCP) and resulted in a more efficient resource allocation. However, PFCC is challenged by a person-centered framework, as children with HMV are treated for various underlying diseases (McCormack and McCance, 2006; Ottonello et al., 2016; Pekcan et al., 2010; Preutthipan, 2015; Reiter et al., 2011).

### Lack of Evidence Concerning QoL in Children with HMV

Little is known on how HMV affects the patient's quality of life (QoL), especially children's (Bergeron, Duggins, Cohen & Ishman, 2018). A longitudinal study from US evaluated a program designed to transition hospitalized children with mechanical ventilation safely home. The results show that the caregivers experienced less anxiety, more time for family activities, less financial burden and improvement of their child's QoL (Schesser, Brodarcki & Lebet, 2020). In situations where the child cannot complete the questionnaire by him- or herself, parents or caregivers can either assist the child in completing the questionnaire, or they can complete the questionnaire for the child. The latter is called a parent-proxy (Hsiao & Nixon, 2008; Lumeng, Warschausky, Nelson & Augenstein, 2001). Due to the underlying diseases of the child receiving HMV, not everyone can complete a questionnaire by him- or herself, therefore parent-proxies sometimes are used (Bergeron, Duggins, Cohen & Ishman, 2018). As treatment with HMV for children increases there is a need to explore the life situation and QoL in children treated with HMV. Further, a life with HMV means technological challenges, high-risk situations, specialized care and knowledge, all affecting the life situation and QoL of the child. Therefore, there is a need to enhance knowledge in the area.

## Aim

The aim of this scoping review was to explore research regarding QoL and lived experience of children and adolescents with home mechanical ventilation.

## Research Question

What is the quality of life in Children with HMV?

## Method

### Study Design

**Figure 1.**
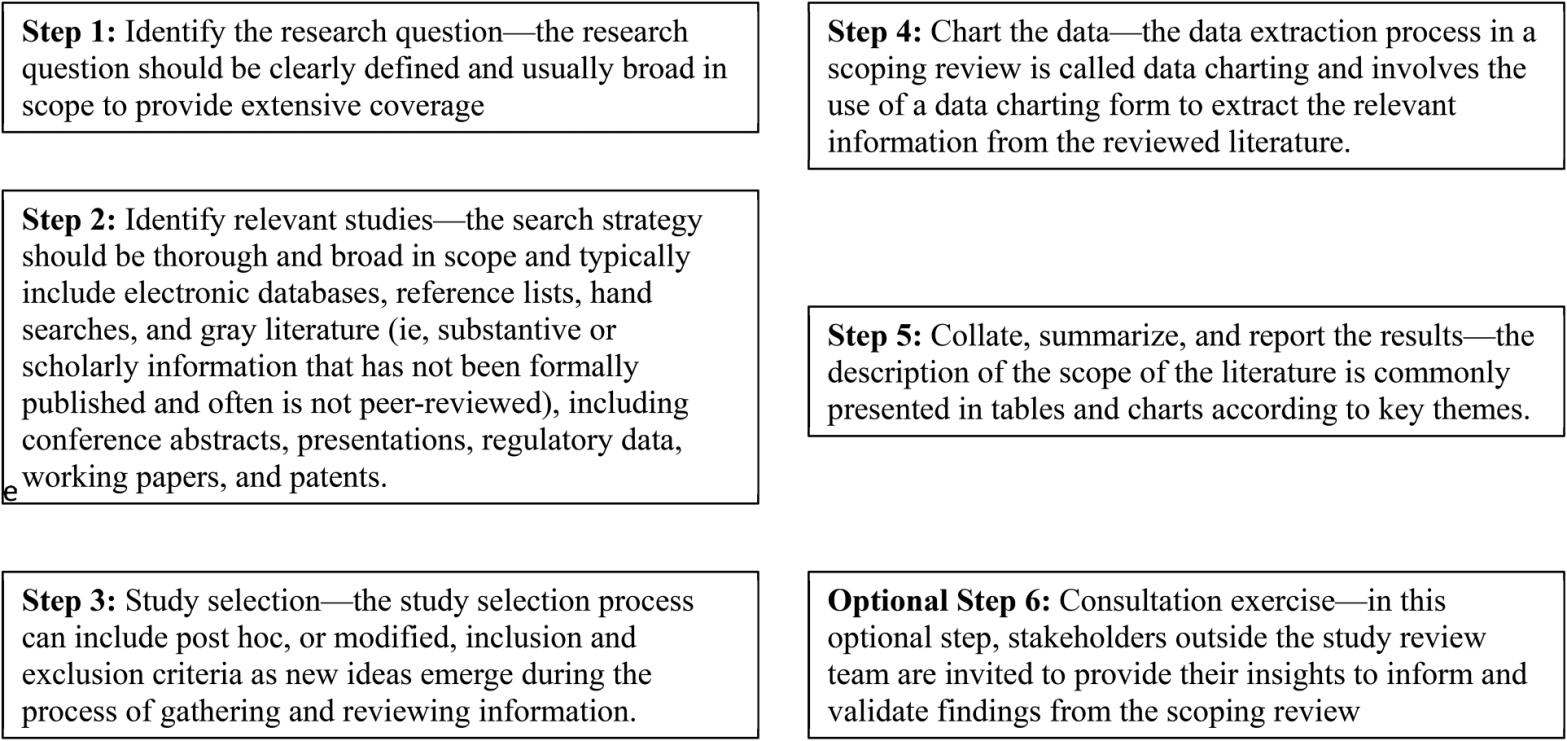
Framework of scoping review as described by Arksey and O’Malley (2005).

The study is a scoping review and data was retrieved and analyze as described by Sucharew and Macaluso (2019). According to Arksey and O’Malley (2005) a scoping review is suitable when the information on a topic has not been comprehensively reviewed or is complex and diverse, or the aim is to identify and map the available evidence (Munn, Peters, Stern, Tufanaru, McArthur, & Aromataris, 2018). We followed the first five steps out of six in the scoping review framework described by Arksey and O’Malley (2005), see Figure 1.

### Inclusion Criteria

In accordance with the first step formulated by Arksey and O’Malley (2005), a research question was formulated and inclusion criteria for the database searches was formulated; children and adolescents aged 0 to 18 years living with HMV, QoL, peer-reviewed original articles published between the years 2000–2020, parent-proxy, and articles written in English.

All articles included in this review had ethical approval and fulfilled the requirements for research studies according to World Medical Association (WMA, 2018).

The exclusion criteria were nurse perceptions’ of children with HMV, adults with HMV and their QoL, parents’ QoL exclusively and data from pediatric hospital care.

### Search Strategy and Data Collection

**Figure 2.**
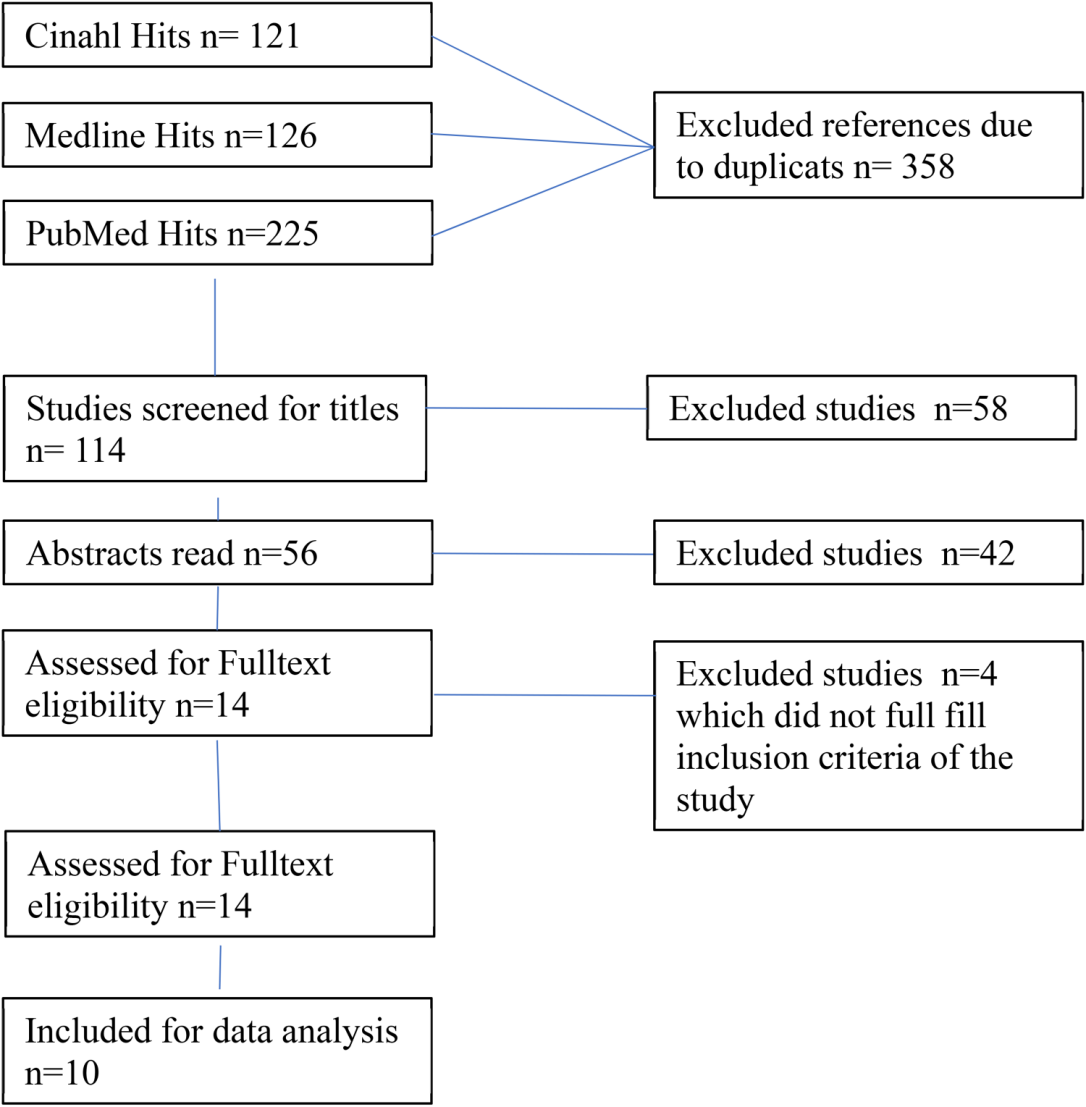
Database search flowchart.

In the second step of the scoping review framework, as described by Arksey and O’Malley (2005) and Sucharew and Macaluso (2019), literature searches were performed. We decided to search the databases: CINAHL, MEDLINE, and PubMed. In the exploratory searches in CINAHL, MEDLINE, and PubMed we used the key words: “Home mechanical ventilation”. Additional keywords words used in the searches were “home mechanical ventilation,” “quality of life,” “children,” “pediatric,” and “children's quality of life”. To achieve a comprehensive search, we used CINAHL headings with the function explode (“ + ” in the search matrix), which resulted in a broad search result compared to their own collected keywords. The keywords from CINAHL headings were QoL + AND (Child + OR Adolescence + ) AND respiration, artificial + . In Medline, the we used a similar function through MeSH, which rendered the keywords QoL AND (Infant + OR Child + OR Adolescent) AND Respiration, artificial + . In PubMed, the keywords from CINAHL headings were used. Manual searches were also performed in accordance with Arksey and O’Malley (2005) and Sucharew and Macaluso (2019), from the selected articles’ reference lists were performed but no articles were included for analysis. In accordance with step three, all database search results were carefully reviewed and articles that corresponded to the research question were chosen for data analysis. In total, 10 articles were chosen for further data analysis and quality assessment. The search strategy and selection is presented in Figure 2.

### Data Analysis

In step four, data was retrieved from the articles and was put in charts and analyzed with thematic analysis as described by Arksey and O’Malley (2005), Sucharew and Macaluso (2019) Polit and Beck (2017). A thematic analysis relies on the similarity and contrast principle. The similarity principle focuses on finding similarities in content, symbols, or meaning. On the other hand, the contrast principle focuses on how content, symbols, or meaning may differ.

The articles were initially read through individually by the present authors to identify how QoL was described by parents and children. In order to get comprehensive results, the authors used colors to highlight the themes in the articles. Thereafter, the authors analyzed the articles to find similarities and differences which formed the result. During this process, the authors identified themes that were discussed and verified. Quotes from the articles formed this review's central themes. These, in turn, were further analyzed to form the result. Both qualitative and quantitative articles were analyzed with the same method. Polit and Beck (2017) declare that this is the method of choice when conducting a literature review and describe the importance of evaluating an article step by step to understand the quality. To assess the quality of the articles included, the authors used assessment tools from Swedish Agency for Health Technology Assessment and Assessment of Social Services (SAHTAASS) (SBU, 2019), this is done to improve the trustworthiness, credibility, and authenticity of the research conducted. In step five, we organized the results according to the emerged themes. The articles were organized in tables depending on the themes. As we had a broad search approach and parent-proxy is frequently used in studies including children and there are scarce research in the field, we decided to include studies where parents’ perception were included.

## Results

Ten studies were selected to the result after data collection and quality assessment evaluation. In Table 1 all the included studies are displayed and summarized. The studies origin from USA, UK, Brazil, Spain, and Canada. From the analysis, the authors derived four themes, *Children's self-reported QoL*, *Parents’ perception and parent-proxy report, Differences between the child’s and parent’s perception,* and *Challenges in daily life.* In Table 2, the included studies are presented within the themes.

**Table 1.** Studies Included in the Review.

Authors, Titles, Journal, Year, Country	Aim	Design	Method		Results	Quality
de Oliveira, F. S., Vasconcelos, V. M., Martins, M. C., & Lúcio, I. M. L. Care to Child with Muscular Dystrophies Dependent of Home Technology: Mothers’ Conception. *Rev Rene,* 2013, Brazil	To understand mothers’ experience of caring for a technology-dependent child and their perceptions’ of the child's QoL.	Descriptive study with a qualitative approach	Data selection: 11 mothers of children with Muscular Dystrophy, aged between 19 and 40 years Data collection: Semi-structured interviews Data Analysis: Referential thematic analysis		Mothers express the negative impact that respirators and other technological devices related to HMV have on their own and the children's QoL. Both anxiety and fear of death are factors that have a direct impact on the QoL.	Moderate quality
González, R. et al. Quality of life in home-ventilated children and their families. *European Journal of Pediatrics* 2017, Spain	To assess perceived QoL in children on HMV and their families and explore problems in their daily life related to HMV.	Cross-sectional study with qualitative and quantitative approach	Data Selection: 41 children aged 1 month to 18 years, with their parents/caregiver Data collection: Semi-structured interview questionnaires and evaluation of perceived QoL by patients and their family (PedsQL 4.0) Data Analysis: Semi-quantitative analysis.		All the participating families were content with the care they gave their children, still they witnessed about how emotionally overwhelming the situation was.	High quality
Graham, R., Rodday, A., & Parsons, S. Family-Centered Assessment and Function for Children With Chronic Mechanical Respiratory Support. *Journal of Pediatric Health Care* 2014,USA	To assess parents’ HRQoL when caring for a child with CRI being treated at home.	A quantitative cross-sectional study	Data Selection: 86 parents/caregivers of children home-based management of CRI. Data Collection: CHRI questionnaire together with global HRQL scale (Global-5). Data analysis: The response for the parents were analysed with Wilcoxon rank sum test or x2 test		Although the children's global HRQoL score was low (63.1, SD = 24.9), the score of “family life” were higher (73.8, SD = 26.5). In parents, both these measures were low.	High quality, low risk for bias
Mah, J., Thannhauser, J., Mcneil, D., & Dewey, D. Being the lifeline: The parent experience of caring for a child with neuromuscular disease on home mechanical ventilation. *Neuromuscular Disorders* 2008, Canada	Parents’ experiences living with a child treated with HMV	Phenomenological study with qualitative approach	Data Selection: 15 mothers and 4 fathers of children with HMV Data Collection: Semi-structured interviews in the participants homes Data Analysis: Downloaded into NVivo 7.0^TM^ (qualitative computer software) for analysis. Subcategory for coding, then topic and coding to identify themes.		Parents express feelings of being the lifeline for their child's life and QoL. The initiation of HMV in their child resulted in changes in their daily lives. Changes, that over time became the new ‘normal’.	High Quality
Noyes, J. Health and quality of life of ventilator-dependent children. *Journal of Advanced Nursing* 2006, UK	Children's lived experiences on QoL and living with HMV, and their parents’ experiences.	Heideggerian phenomenology with qualitative approach.	Data Selection: 53 children and their parents Data collection: Semi-structured interviews in the participants homes Data analysis: Tape recorded, transcribed and anonymized. “Framework” approach for analysis (guiding). Heidegger's circle for themes analysis in order to get correct descriptions participants experiences.		The themes gave some common features in all participants. The children felt better when they had the ventilation, they had enough air for breathing, rendering a better QoL. It was not possible to get an understanding on how much more they gained in QoL, especially amongst those who could not communicate by speech or those with severe neurological impairment.	High Quality
Noyes, J. Comparison of ventilator-dependent child reports of health-related quality of life with parent reports and normative populations. *Journal of Advanced Nursing* 2007, UK	To report ventilator-dependent children's and their parents’ perceived HRQoL using a validated instrument.	Case study with a quantitative approach.	Data Selection: 35 ventilator-dependent children and their parents. Data Collection: KINDL questionnaire was used to measure various dimensions of QoL. Data Analysis: Descriptive statistics were determined from all variable		The ventilator-dependent children who participated in this study reported a significantly lower HRQoL overall, compared to school children.in general. Parents and children rated the children's overall HRQoL the same but parents reported lower scores on their child's underlying disease and relation with friends	Moderate Quality, bias may exist
Rodday, A. M., Graham, R. J., Weidner, R. A., Terrin, N., Leslie, L. K., & Parsons, S. K. Predicting Health Care Utilization for Children With Respiratory Insufficiency Using Parent-Proxy Ratings of Children's Health-Related Quality of Life. *Journal of Pediatric Healthcare* 2017, USA	To determine if parent-proxy reports of children's HRQoL affected future health care utilization. Furthermore, to get an understanding if these parent-proxy reports contribute with additional information that may help predicting future health care needs	Longitudinal study with quantitative approach	Data Selection: 140 patients aged 30 days to 22 years, and their parents/caregivers. Data Collection: CHRI questionnaire with both child and parent version was used to collect parent-proxy reports. Data Analysis: To identify differences in the responds regarding various of factors Wilcoxon rank sum test and chi-square test were used		Three quarters of the participating children utilized healthcare and 32% were hospitalized. Children who reported poor/fair global HRQoL had 3.7 times more documented days in hospital care compared to those who reported good/excellent global HRQoL.	High quality, low risk for bias
Sarvey, S. I. Living with a machine: the experience of the child who is ventilator dependent. *Issues in Mental Health Nursing* 2008, USA	To obtain and reflect the first-person perspective of children's experience of being ventilator-dependent.	Phenomenological interviews with a qualitative approach.	Data Selection: 9 children who were ventilator-dependent for at least eight hours eper day. Data Collection: Phenomenological interviews that was tape-recorded and transcribed verbatim.. Data Analysis: The collected data was analysed using a hermeneutic method, which generated a thematic structure.	Common for all the participating children was a feeling of never being alone. They expressed feelings of being dependent on both the people who cared for them and the machines that kept them alive.		Moderate quality
Seear, M., Kapur, A., Wensley, D., Morrison, K., & Behroozi, A. The quality of life of home-ventilated children and their primary caregivers plus the associated social and economic burdens: A prospective study. *Archives of Disease in Childhood* 2016, Canada	To assess the impact of home ventilation in children and their QoL.	A prospective study with a quantitative approach.	Data Selection: 90 families with a child treated with home ventilation. Data Collection: PedsQL were used to measure QoL in the participating children. Caregiver Impact Scale (CIS) were used to measure how the main caregiver was affected by home ventilation. Data was collected via telephone after one and two months. Data Analysis: All collected data were managed using non-parametric methods.	Most of the participating children and families had active lives, such as wheelchair sports and vacations abroad. The burden of care was reported as mild/moderate by over 90% of the participants. Among the primary caregivers, nearly 25% reported the burden of care as severe.		High quality, low risk for bias
Spratling, R. The experiences of medically fragile adolescents who require respiratory assistance. *Journal of Advanced Nursing* 2012, USA	Respiratory assistant adolescents experiences on their own life.	Interpretive phenomenology study with qualitative approach.	Data selection: 11 adolescents from southeast USA Data collection: Semi-structured interviews with adolescents in their homes Data Analysis: Audiotaped and transcribed verbatim. The interviews were summarized, coded and evaluated for emerging themes. Found themes were validated with participants and within the research team.	Five themes emerged from the interviews: ‘Get to know me’, ‘Allow me to be myself’, ‘Being there for me’, ‘No matter what, technology helps’ and ‘I am an independent person’.		High quality

**Table 2.** Theme Matrix.

Author	Children's self-reported QoL	Parents’ perception and parent-proxy report	Differences between the child's and parents’ perception	Challenges in daily life
de Oliveira et al. (2013)		X		X
González et al. (2017)	X	X	X	X
Graham et al. (2014)		X	X	
Mah et al. (2008)		X		X
Noyes (2006)	X	X	X	
Noyes (2007)	X	X	X	
Rodday et al. (2017)		X		
Sarvey (2008)	X			X
Seear et al. (2016)		X	X	X
Spratling (2012)	X			X

### Children's Self-Reported QoL

An important finding was that children expressed that knowledge about their condition and speaking their minds were essential to them, so other people could understand that they had a good life despite the HMV (Sarvey, 2008). It is most important to let children with HMV speak their minds, answer for themselves and express their feelings, as well as assess their own QoL (Noyes 2006; Noyes 2007; Sarvey 2008; Spratling 2012). Furthermore, the perception of QoL involves more than the technology itself; children take more into account, such as going to school, meeting friends, and having a social life. They report that limitations of the “machine” have a significant impact on the daily life. Children would also like to be seen as human beings rather than the machine helping them to breathe (Sarvey 2008; Spratling 2012).

Being able to breathe properly was important for the children and doing things they wished to do increased their QoL. Children did not perceive the ventilator as a restriction, but some mentioned that they would like to be without it. Participating in activities increased their QoL (Noyes, 2006). However, they sometimes felt limited by the machine, and felt that they could not be with friends which affected them negatively (Sarvey, 2008).

The children's perception of QoL was depending on when the HMV was initiated and how long they have been on HMV. Children, that had been on HMV from infancy, had adapted to the situation and the ventilator did not affect their QoL. Children with HMV did not display a lower QoL compared to children in their surroundings. They had a good life (González et al., 2017; Noyes, 2006). However, it is important to note that older children on HMV experienced a lower in QoL, which can be related to a late onset of the disease, or a severe injury that had led to initiation of HMV. They could have memories from before and talked about getting well again. They additionally expressed that the society stopped them from being fully included and expressed feelings of being left out, which affected their QoL negatively, followed by depression. The perceived QoL among children and adolescents with HMV was in general lower compared to healthy children and children with other chronic diseases (Noyes 2007; González et al. 2017). Noyes (2007) reported that HMV children had a total QoL score of 62.40, whereas healthy children scored 76.75. This confirms that QoL in children with HMV was lower than among healthy children, supported by González et al. (2017).

### Parents’ Perception and Parent-Proxy Report

#### Parent’s perception

Home Mechanical Ventilation makes it possible for children with various kinds of conditions to be cared for in their home environment (González et al., 2017; Graham et al., 2014; Mah, Thannhauser, McNeil & Dewey, 2008; Noyes 2006; Rodday et al., 2017; Seear, Kapur, Wensley, Morrison & Behroozi, 2016). However, when the child is transferred from specialized wards to their home, pressure and demands are put on the primary caregiver in the home environment, usually one of the parents and specifically the mother. The pressure and demands laid upon the mothers could, in turn, affect the QoL of their children (de Oliveira et al., 2013; Graham et al., 2013; Seear et al., 2016).

Parents were concerned about their child's QoL, and they did their best to verify that the child had some sort of happiness in their life that was not limited to the child's cognitive and physical ability (Mah et al., 2008). Most parents reported their child's QoL to be good. However, they expressed concerns about the future. Some parents had fought for their children and noticed an increased QoL. These parents had done extensive research on their own about HMV because they thought it could help their child in living a more normal life, even with a severe medical condition (Noyes, 2006). Parents talked about the high responsibility they had to undertake when their child was initiated with HMV (Mah et al., 2008). They expressed that health care staff should know how to introduce HMV to parents. Some parents felt pushed by them to initiate HMV in their child. In order to make well-founded decisions regarding HMV, parents felt that information from healthcare staff about HMV should be evidence-based and non-biased (a.a.). HCP must evaluate the need for support in this group of patients to increase QoL of the child and avoid admittance to the hospital due to preventable complications. By having close contact with the parents, necessary adjustments can easily be made to the ventilator (Rodday et al., 2017).

#### Parent-proxy report

Regarding the very young children, the parent-proxy were essential. They said that these children were cared for and loved and they were trying to give them as good QoL as possible (Mah et al., 2008; Noyes, 2006). When asked, these parents would make the same decision to initiate HMV again. Even though HMV meant an increased workload on the families, it meant a prolonged life and increased the child's QoL (Mah et al., 2008). However, parents rated their child's QoL lower than parents with healthy children or children with other chronic diseases. When compared to scores of those with chronic diseases, children with HMV score lower in all dimensions except for emotional functioning (EF) and school functioning (SF) (González et al., 2017). The severity of the child's condition had a negative impact on the parent-proxy scoring (Graham et al., 2013). Despite this, the feeling of hope among parents was present. The hope for a future treatment or even a cure (de Oliveira et al., 2013).

### Differences Between the Child's and Parents’ Perception

There were differences between parents’ and children's answers related to QoL, where children, in some cases, rated higher than parents on questions and vice versa. The cases where the parents rated higher than children were in general few. Overall, the parents rated their child's QoL lower than the child itself (González et al., 2017; Graham et al., 2013; Noyes 2007; Rodday et al., 2017; Seear et al., 2016). In common, for all studies was that the result showed that children did not perceive their QoL as low as their parents did. This might be associated to not remembering how life was before (Noyes 2006). Some children were not aware of that their social life was any different from others; only the parents saw a difference in the social life and exclusion from society. However, some children were aware of their social life exclusion and felt sad and experienced a lowered self-esteem. In a prospective study about QoL by Seear et al. (2016), the participating children, 42.2%, completed the questionnaire about their own QoL, and 57.8% were assessed by parent-proxy. There was no difference between the child's self-reported QoL and the one reported by parent-proxy (Noyes, 2007; Seear et al., 2016).

### Challenges in Daily Life

In many studies, challenges in daily life were related to the underlying diseases the children had and the treatment of it (de Oliveira et al., 2013; González et al., 2017; Mah et al., 2008; Sarvey 2008; Seear et al., 2016). Most of the children expressed that the major challenges in their daily lives were associated to everyday things, such as school, friends, or other activities, and not to the ventilator (Spratling, 2012; Sarvey, 2008). Due to several experiences of crucial and life-threatening situations at home and the hospital, anxiety and fear of death of the child were often present feelings among parents. The daily life of the children was considered normal in the perception of their mothers. It was, however, stated that some aspects in daily life, such as taking a bath or putting clothes on, could be challenging and requiring the assistance of some kind (de Oliveira et al., 2013).

When a child, depending on medical technology, is discharge to home is a great challenge for the family. Often, the mother has to leave her job and professional commitments to stay at home, which is affecting families in various ways, not at least financially. The mothers are vulnerable to stress and anxiety, being in a situation where they have less control. It requires skills to care for a chronically ill child. Nurses could support the family in changing their perspectives on the child's disease and teach them how to cope with the situation (de Oliveira et al., 2013).

Adolescents with HMV expressed that they did not want to be seen as patients requiring respiratory assistance by the nurses who treated them. They wanted the nurses to treat them as normal people, ask them personal questions, and get to know them as individuals (Spratling, 2012). Nurses could help destress the underlying disease by making them focus on different aspects of their day-to-day lives. One adolescent participating in the study described that she was more comfortable being treated when nurses got to know her. Spratling (2012) reports that despite the technology being a part of adolescents’ daily lives, their focus was instead on “normal” things, such as school, friends, and family. They were aware of how technology helped them in their daily lives. These technological devices were perceived as something that helped improve their QoL, rather than something limiting them. Despite being dependent on respiratory assistance, adolescents had an urge to become independent human beings. One participating adolescent described that he independently functioned in school, even with respiratory assistance (Spratling, 2012).

## Discussion

This study aimed to explore research about quality of life in home mechanical ventilated children and adolescents, which includes their parents’-, or caregivers’ perception of the child's quality of life. The result shows that QoL of children with HMV is lower than of the general population and other chronically ill children. Parents seem to rate their child's QoL lower than the child itself, especially regarding social life and emotional dimensions. Further, HMV does not only affect the treated child or adolescent but the whole family. The articles included in this study, have a wide geographical distribution, i.e., Europe, North- and South America.

### QoL in Children with HMV

The result of the present study shows that there are scarce research in the field and that children with HMV display a lower QoL than the general population or then children with other chronic diseases. These findings are supported by Cunha et al. (2013), who found that QoL among children in PICU was lower than other children and children with chronic diseases. Despite this, some children with HMV did rate their QoL lower than children in general in the same age group. For them, life was just like anyone else's (Noyes, 2006), and one essential factor of how children on HMV perceived their QoL was the age when HMV was initiated. If HMV stared in early life, the child rated their QoL higher than those who had memories of their lives before HMV. This may explain a lower QoL in older children and adolescents. However, in a study by Israelsson-Skogsberg, Hedén, Lindahl, & Laakso, 2018, children did not see themselves as sick, except when they got ill. They felt as well happy when joining in everyday activities such as school and socializing with friends.

Cunha et al. (2013) suggest that QoL should be measured at baseline. This would allow HCP to evaluate how the treatment affects the child's QoL while undergoing HMV and give researchers statistical data on how the QoL changes over time. Rodday et al. (2017) further claim QoL to be useful for nurses. By regularly evaluating QoL among children with HMV, nurses would detect decreased QoL, and necessary treatment adjustments could be inducted

### Parent Proxy Reported QoL

In some of the studies (de Oliveira et al., 2013; González et al.; Mah et al., 2008; Noyes 2006; Noyes 2007; Rodday et al., 2017; Seear et al., 2016) the child could not, due to his or her condition, independently complete the questionnaire. In these cases, a primary caregiver or a parent, so called parent-proxy, completed the questionnaire or interview for the child. This is a common method used in research as described by Hsiao & Nixon, 2008; Lumeng, Warschausky, Nelson & Augenstein, 2001. Our review shows that there is a discrepancy on how children their QoL compared to how their parents rate and describe the child's QoL. Overall, parents report their child's QoL lower than the child does, that also is stated by González et al. (2017), Mah et al. (2008), Noyes (2007), and Rodday et al. (2017). Vieira and Linhares (2016) found differences between self-reports and parent proxy reports in a systematic review on QoL among children born preterm. Furthermore, parents’ experiences when caring for their child at home might influence the parent-proxy reports (de Oliveira et al., 2013; González et al., 2017; Rodday et al., 2017). Parents showed a decreased QoL, which also might influence the outcome, confirmed by Mah et al. (2008) and Seear et al. (2016). However, children tended to rate their QoL higher than their parents did, in line with the present study results.

### Family- and Caregivers’ Perspective

The results revealed that children and adolescents with HMV meet nurses and HCP daily, who need to be aware of the child's needs, both physical and emotional (de Oliveira et al., 2013; Spratling, 2012). By implementing an approach that focuses on aspects that increase the patient's QoL, nurses and caregivers could meet their needs more person-centered. McCormack and McCance (2006) presented a framework in which adequate care is based on an equal partnership between the nurse, patient, and family, which supports this. They are optimizing daily routines and activities with a person-centered approach to give every patient the best possible QoL based on their individual preferences (Israelsson-Skogsberg et al., 2019; McCormack & McCance, 2006; Spratling, 2012). The possibility of having assistance, gives the child and adolescent with HMV an opportunity to have activities outside home or hospital which increases the QoL (Israelsson-Skogsberg et al., 2018). Siblings felt gratified having their brother or sister at home and not in the hospital. However, some situations troubled them, such as not having the possibility to do some activities together as siblings because HMV limited the sibling (Israelsson-Skogsberg, Markström, Laakso, Hedén & Lindahl, 2019).

Another factor that can influence the QoL of children with HMV is the high costs which could be a burden to the family. Depending on the healthcare system or covering of the insurance, parents might have to finance the HMV and assistance themselves (Preutthipan, 2015). This could affect the QoL negatively (de Oliveira et al., 2013; Graham et al., 2013; Seear et al., 2016). Furthermre, it is important that the home transfer is carefully planned and that all involved parts are familiar with the goals. This will lower the stress for the families (Ertugrul, Baykaci, Ertugrul, Kesici & Yalcin, 2017; Ottonello et al., 2007). This review reveales a need for a well-established system for discharge. Such systems will assist families during home transfer and should include follow-up plans for evaluation of the HMV, additional support if needed, in order to increase the child's QoL (de Oliveira et al., 2013; Graham et al., 2013; Seear et al., 2016). The results also revealed a need for standardized routines and guidelines for HMV and support from specialized teams to support the child and the families, especially when the child was ill and in need of hospital care. This is confirmed by research by Falkson, Knecht, Hellmers, and Metzing (2017) and Ertugrul et al., (2017).

### Methodological Considerations

Due to limited research in the field, a scoping review was chosen as method and we used the framework stated by Sucharew and Macaluso (2019). According to Arksey and O’Malley (2005). This review is small due to time and financially resources but implicates the need for further studies in the field. To increase the reliability of the results the authors read, analyzed, and assessed the articles individually and then compared their findings to create the result. To further enhance the trustworthiness, quality assessment tools were used to verify the quality of each included article. The transferability of the results, can be discussed due to the limited amount of articles and the lack of studies from low- and middle income countries. But the studies were quality assessed and had different research methods which enhance the transferability of the results.

## Conclusions

This is the first literature review focusing on HMV in the pediatric population. The main finding was that children with HMV rate their overall QoL lower compared to the general population or to children with other chronic diseases. HMV does not only affect the treated child or adolescent but also the whole family. It is important to regularly measure and evaluate QoL in children and adolescents with HMV to provide person-centered care and increase their QoL and ease the burden of their families. There is a lack of validated pediatric HMV specific QoL tools and evidence regarding QoL goals in the pediatric HMV population.

### Implications for Clinical Practice

It is important to regularly measure and evaluate QoL in children and adolescents with HMV to provide person-centered care to increase their QoL. This study also shows a need for education in the area to enhance knowledge and thereby prevent complications and quality of life and to carefully plan the goals regarding HMV in the peadiatric population.
